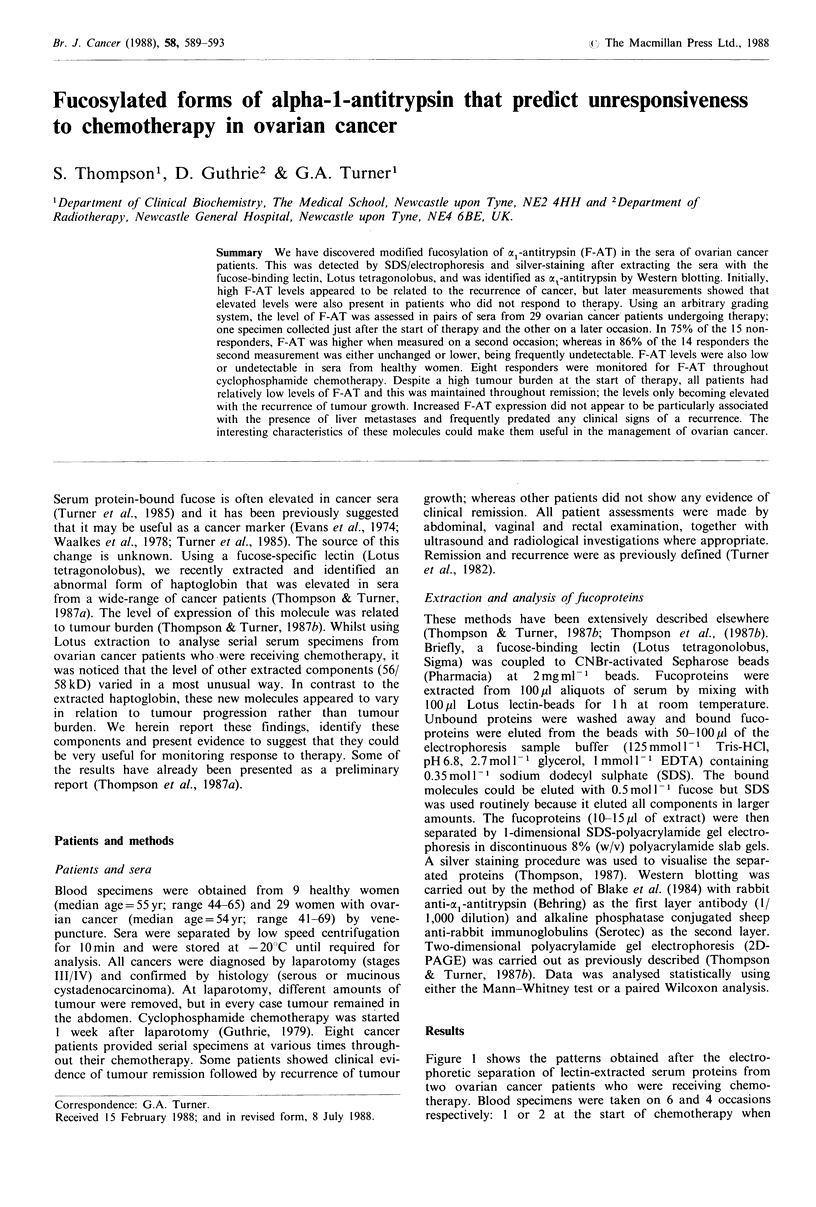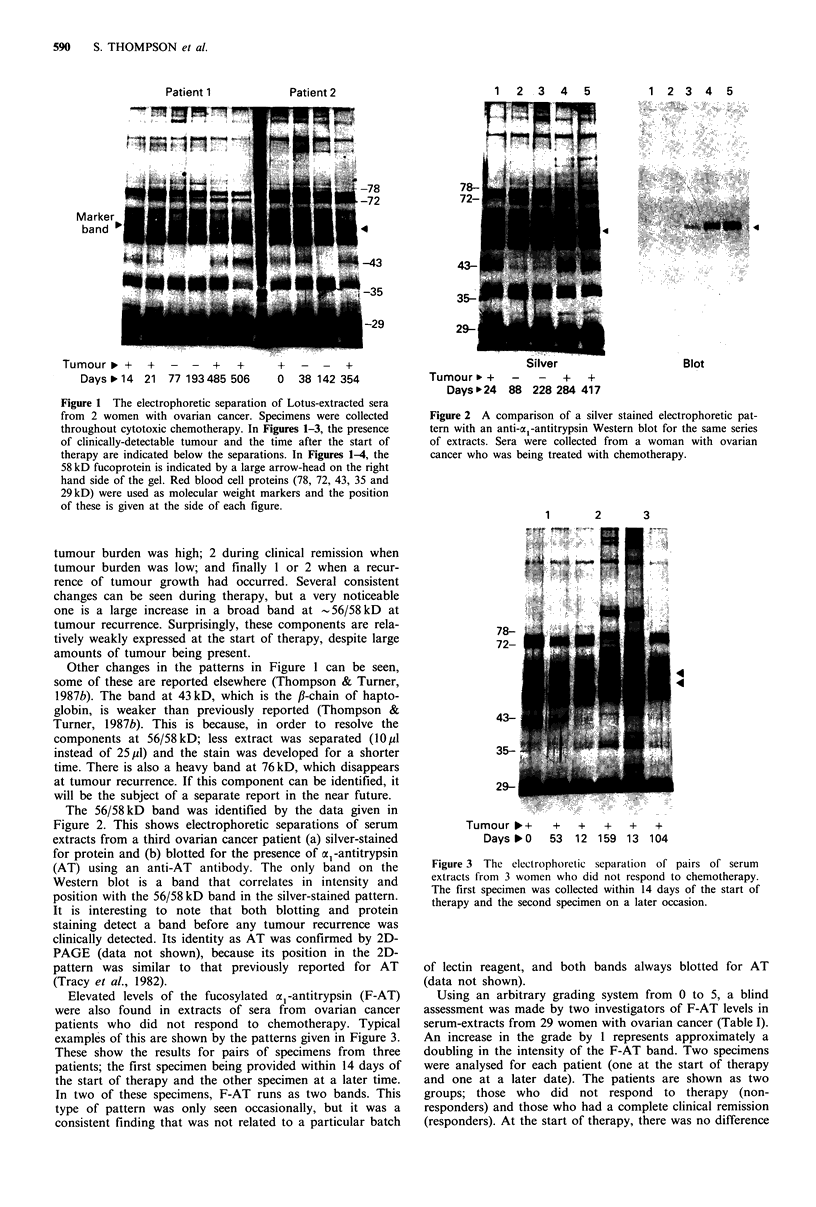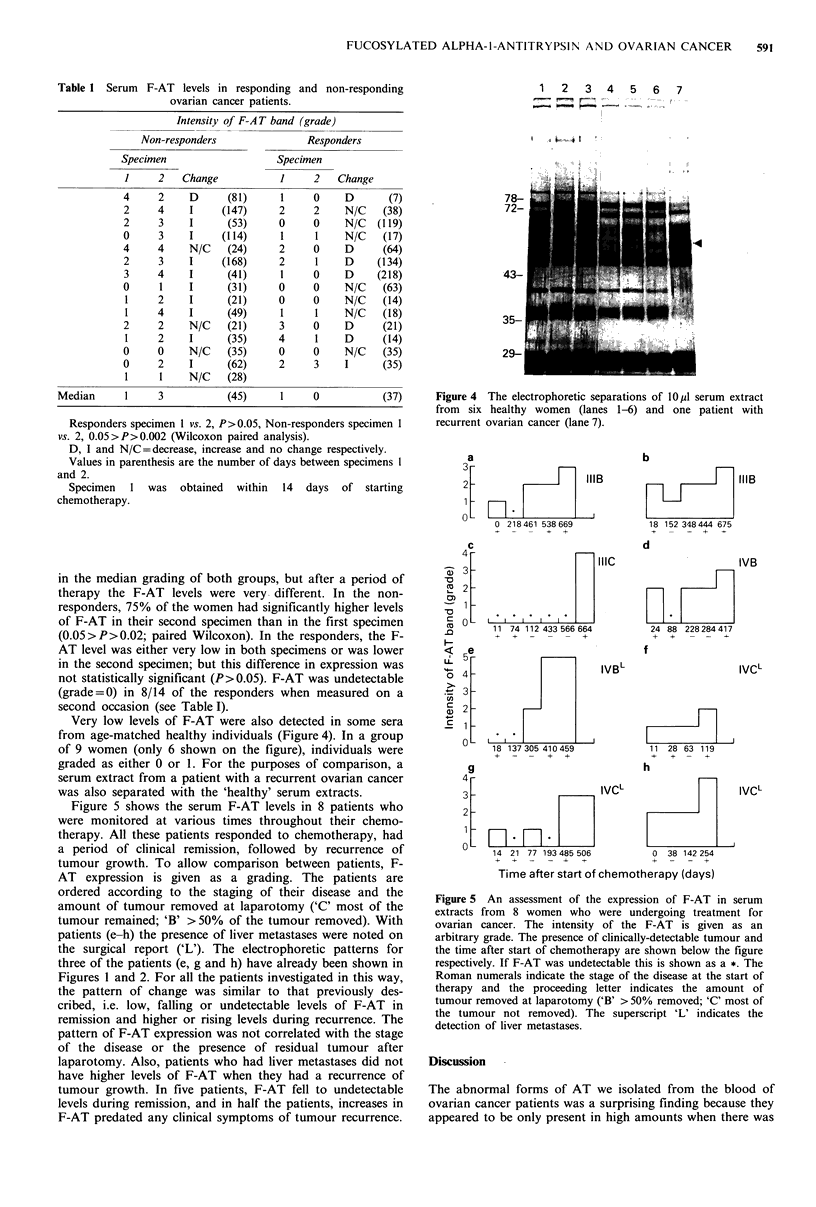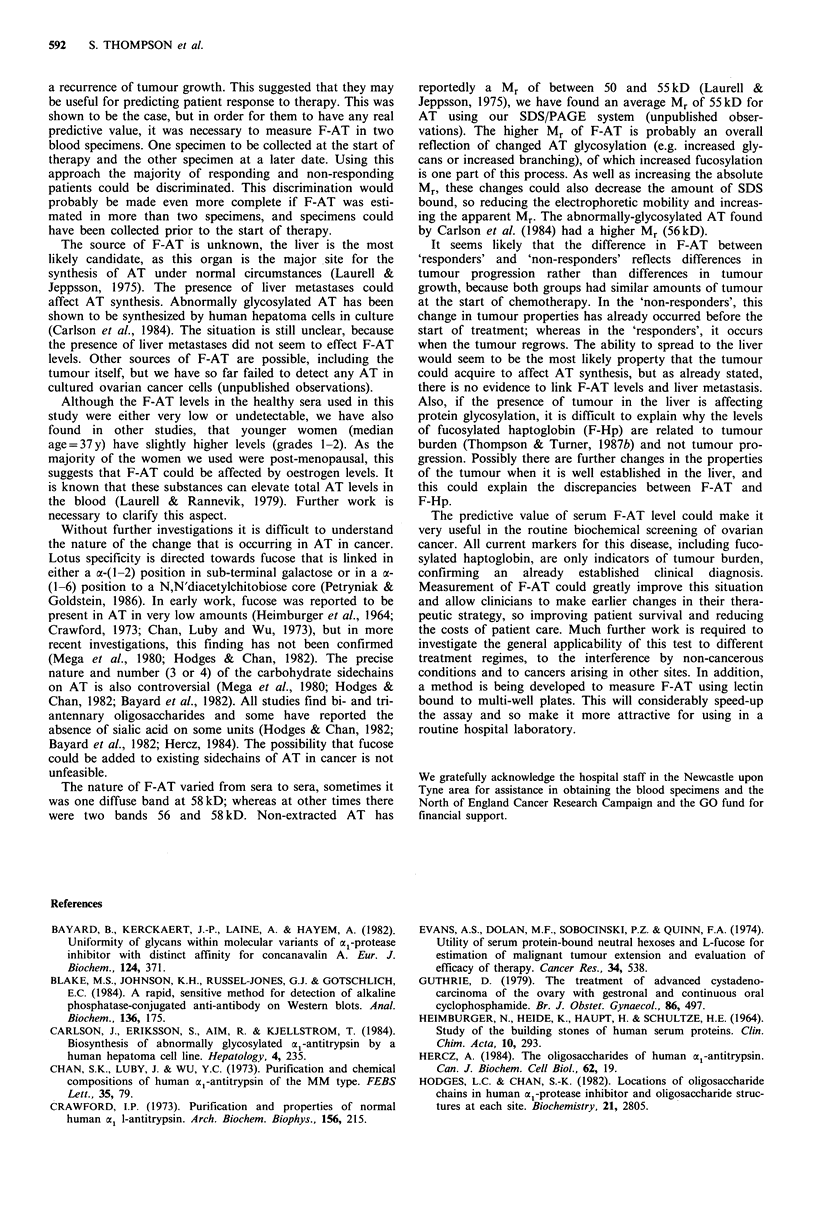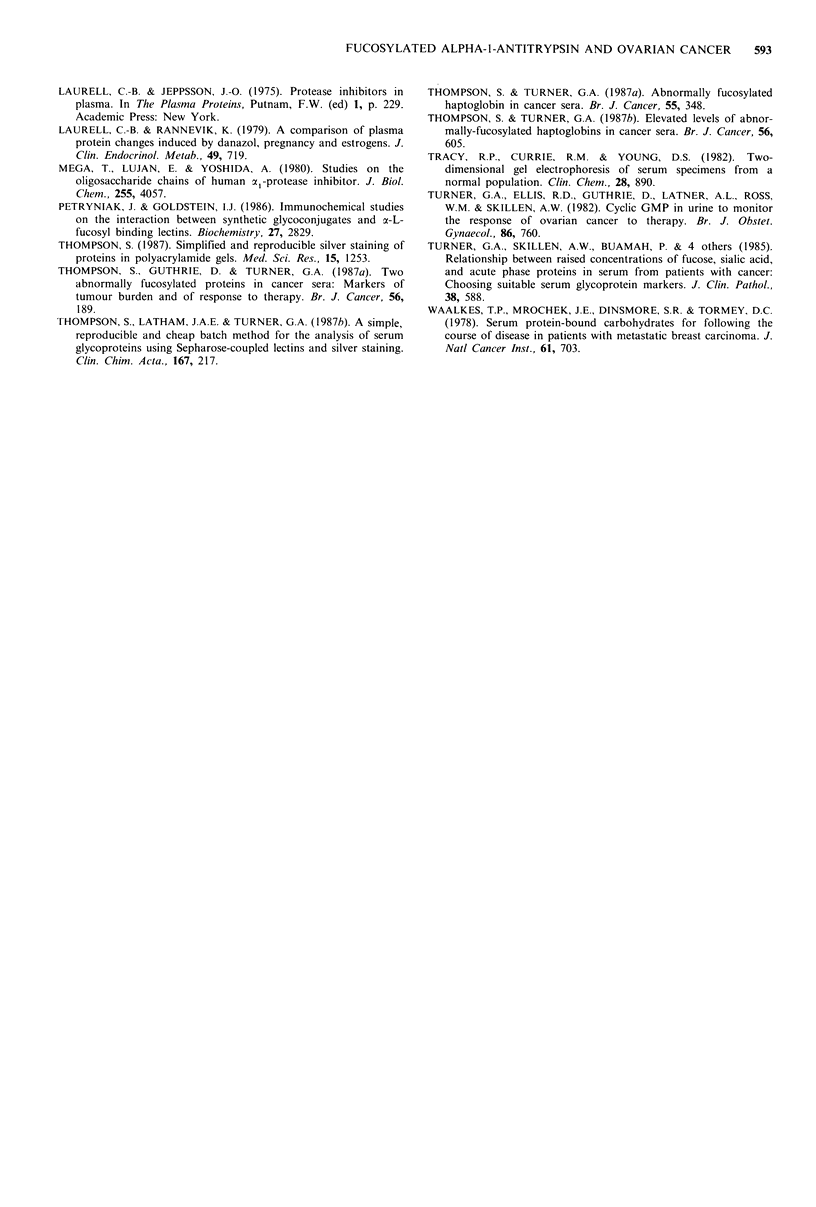# Fucosylated forms of alpha-1-antitrypsin that predict unresponsiveness to chemotherapy in ovarian cancer.

**DOI:** 10.1038/bjc.1988.265

**Published:** 1988-11

**Authors:** S. Thompson, D. Guthrie, G. A. Turner

**Affiliations:** Department of Clinical Biochemistry, Medical School, Newcastle upon Tyne, UK.

## Abstract

**Images:**


*Br. J. Cancer* (1988), **58**, 589–593 © The Macmillan Press Ltd., 1988

### Fucosylated forms of alpha-1-antitrypsin that predict unresponsiveness to chemotherapy in ovarian cancer

S. Thompson[1], D. Guthrie[2] & G.A. Turner[1]

[1]*Department of Clinical Biochemistry, The Medical School, Newcastle upon Tyne, NE2 4HH and* [2]*Department of Radiotherapy, Newcastle General Hospital, Newcastle upon Tyne, NE4 6BE, UK.*

**Summary** We have discovered modified fucosylation of $\alpha_1$-antitrypsin (F-AT) in the sera of ovarian cancer patients. This was detected by SDS/electrophoresis and silver-staining after extracting the sera with the fucose-binding lectin, Lotus tetragonolobus, and was identified as $\alpha_1$-antitrypsin by Western blotting. Initially, high F-AT levels appeared to be related to the recurrence of cancer, but later measurements showed that elevated levels were also present in patients who did not respond to therapy. Using an arbitrary grading system, the level of F-AT was assessed in pairs of sera from 29 ovarian cancer patients undergoing therapy; one specimen collected just after the start of therapy and the other on a later occasion. In 75% of the 15 non-responders, F-AT was higher when measured on a second occasion; whereas in 86% of the 14 responders the second measurement was either unchanged or lower, being frequently undetectable. F-AT levels were also low or undetectable in sera from healthy women. Eight responders were monitored for F-AT throughout cyclophosphamide chemotherapy. Despite a high tumour burden at the start of therapy, all patients had relatively low levels of F-AT and this was maintained throughout remission; the levels only becoming elevated with the recurrence of tumour growth. Increased F-AT expression did not appear to be particularly associated with the presence of liver metastases and frequently predated any clinical signs of a recurrence. The interesting characteristics of these molecules could make them useful in the management of ovarian cancer.

Serum protein-bound fucose is often elevated in cancer sera (Turner *et al.*, 1985) and it has been previously suggested that it may be useful as a cancer marker (Evans *et al.*, 1974; Waalkes *et al.*, 1978; Turner *et al.*, 1985). The source of this change is unknown. Using a fucose-specific lectin (Lotus tetragonolobus), we recently extracted and identified an abnormal form of haptoglobin that was elevated in sera from a wide-range of cancer patients (Thompson & Turner, 1987*a*). The level of expression of this molecule was related to tumour burden (Thompson & Turner, 1987*b*). Whilst using Lotus extraction to analyse serial serum specimens from ovarian cancer patients who were receiving chemotherapy, it was noticed that the level of other extracted components (56/58 kD) varied in a most unusual way. In contrast to the extracted haptoglobin, these new molecules appeared to vary in relation to tumour progression rather than tumour burden. We herein report these findings, identify these components and present evidence to suggest that they could be very useful for monitoring response to therapy. Some of the results have already been presented as a preliminary report (Thompson *et al.*, 1987*a*).

### Patients and methods

### *Patients and sera*

Blood specimens were obtained from 9 healthy women (median age = 55 yr; range 44–65) and 29 women with ovarian cancer (median age = 54 yr; range 41–69) by venepuncture. Sera were separated by low speed centrifugation for 10 min and were stored at −20°C until required for analysis. All cancers were diagnosed by laparotomy (stages III/IV) and confirmed by histology (serous or mucinous cystadenocarcinoma). At laparotomy, different amounts of tumour were removed, but in every case tumour remained in the abdomen. Cyclophosphamide chemotherapy was started 1 week after laparotomy (Guthrie, 1979). Eight cancer patients provided serial specimens at various times throughout their chemotherapy. Some patients showed clinical evidence of tumour remission followed by recurrence of tumour growth; whereas other patients did not show any evidence of clinical remission. All patient assessments were made by abdominal, vaginal and rectal examination, together with ultrasound and radiological investigations where appropriate. Remission and recurrence were as previously defined (Turner *et al.*, 1982).

### *Extraction and analysis of fucoproteins*

These methods have been extensively described elsewhere (Thompson & Turner, 1987*b*; Thompson *et al.*, (1987*b*). Briefly, a fucose-binding lectin (Lotus tetragonolobus, Sigma) was coupled to CNBr-activated Sepharose beads (Pharmacia) at 2 mg ml$^{-1}$ beads. Fucoproteins were extracted from 100 $\mu$l aliquots of serum by mixing with 100 $\mu$l Lotus lectin-beads for 1 h at room temperature. Unbound proteins were washed away and bound fucoproteins were eluted from the beads with 50–100 $\mu$l of the electrophoresis sample buffer (125 mmol l$^{-1}$ Tris-HCl, pH 6.8, 2.7 mol l$^{-1}$ glycerol, 1 mmol l$^{-1}$ EDTA) containing 0.35 mol l$^{-1}$ sodium dodecyl sulphate (SDS). The bound molecules could be eluted with 0.5 mol l$^{-1}$ fucose but SDS was used routinely because it eluted all components in larger amounts. The fucoproteins (10–15 $\mu$l of extract) were then separated by 1-dimensional SDS-polyacrylamide gel electrophoresis in discontinuous 8% (w/v) polyacrylamide slab gels. A silver staining procedure was used to visualise the separated proteins (Thompson, 1987). Western blotting was carried out by the method of Blake *et al.* (1984) with rabbit anti-$\alpha_1$-antitrypsin (Behring) as the first layer antibody (1/1,000 dilution) and alkaline phosphatase conjugated sheep anti-rabbit immunoglobulins (Serotec) as the second layer. Two-dimensional polyacrylamide gel electrophoresis (2D-PAGE) was carried out as previously described (Thompson & Turner, 1987*b*). Data was analysed statistically using either the Mann–Whitney test or a paired Wilcoxon analysis.

### Results

Figure 1 shows the patterns obtained after the electrophoretic separation of lectin-extracted serum proteins from two ovarian cancer patients who were receiving chemotherapy. Blood specimens were taken on 6 and 4 occasions respectively: 1 or 2 at the start of chemotherapy when

Correspondence: G.A. Turner.
Received 15 February 1988; and in revised form, 8 July 1988.

**Figure 1** The electrophoretic separation of Lotus-extracted sera from 2 women with ovarian cancer. Specimens were collected throughout cytotoxic chemotherapy. In **Figures 1–3**, the presence of clinically-detectable tumour and the time after the start of therapy are indicated below the separations. In **Figures 1–4**, the 58 kD fucoprotein is indicated by a large arrow-head on the right hand side of the gel. Red blood cell proteins (78, 72, 43, 35 and 29 kD) were used as molecular weight markers and the position of these is given at the side of each figure.

**Figure 2** A comparison of a silver stained electrophoretic pattern with an anti-$\alpha_1$-antitrypsin Western blot for the same series of extracts. Sera were collected from a woman with ovarian cancer who was being treated with chemotherapy.

tumour burden was high; 2 during clinical remission when tumour burden was low; and finally 1 or 2 when a recurrence of tumour growth had occurred. Several consistent changes can be seen during therapy, but a very noticeable one is a large increase in a broad band at $\sim$56/58 kD at tumour recurrence. Surprisingly, these components are relatively weakly expressed at the start of therapy, despite large amounts of tumour being present.

Other changes in the patterns in Figure 1 can be seen, some of these are reported elsewhere (Thompson & Turner, 1987*b*). The band at 43 kD, which is the $\beta$-chain of haptoglobin, is weaker than previously reported (Thompson & Turner, 1987*b*). This is because, in order to resolve the components at 56/58 kD; less extract was separated (10 $\mu$l instead of 25 $\mu$l) and the stain was developed for a shorter time. There is also a heavy band at 76 kD, which disappears at tumour recurrence. If this component can be identified, it will be the subject of a separate report in the near future.

The 56/58 kD band was identified by the data given in Figure 2. This shows electrophoretic separations of serum extracts from a third ovarian cancer patient (a) silver-stained for protein and (b) blotted for the presence of $\alpha_1$-antitrypsin (AT) using an anti-AT antibody. The only band on the Western blot is a band that correlates in intensity and position with the 56/58 kD band in the silver-stained pattern. It is interesting to note that both blotting and protein staining detect a band before any tumour recurrence was clinically detected. Its identity as AT was confirmed by 2D-PAGE (data not shown), because its position in the 2D-pattern was similar to that previously reported for AT (Tracy *et al.*, 1982).

Elevated levels of the fucosylated $\alpha_1$-antitrypsin (F-AT) were also found in extracts of sera from ovarian cancer patients who did not respond to chemotherapy. Typical examples of this are shown by the patterns given in Figure 3. These show the results for pairs of specimens from three patients; the first specimen being provided within 14 days of the start of therapy and the other specimen at a later time. In two of these specimens, F-AT runs as two bands. This type of pattern was only seen occasionally, but it was a consistent finding that was not related to a particular batch

**Figure 3** The electrophoretic separation of pairs of serum extracts from 3 women who did not respond to chemotherapy. The first specimen was collected within 14 days of the start of therapy and the second specimen on a later occasion.

of lectin reagent, and both bands always blotted for AT (data not shown).

Using an arbitrary grading system from 0 to 5, a blind assessment was made by two investigators of F-AT levels in serum-extracts from 29 women with ovarian cancer (Table I). An increase in the grade by 1 represents approximately a doubling in the intensity of the F-AT band. Two specimens were analysed for each patient (one at the start of therapy and one at a later date). The patients are shown as two groups; those who did not respond to therapy (non-responders) and those who had a complete clinical remission (responders). At the start of therapy, there was no difference

**Table 1** Serum F-AT levels in responding and non-responding ovarian cancer patients.

| | *Intensity of F-AT band (grade)* | | | | | | |
|---|---|---|---|---|---|---|---|
| | *Non-responders* | | | | *Responders* | | |
| | *Specimen* | | | | *Specimen* | | |
| | *1* | *2* | *Change* | | *1* | *2* | *Change* | |
| | 4 | 2 | D | (81) | 1 | 0 | D | (7) |
| | 2 | 4 | I | (147) | 2 | 2 | N/C | (38) |
| | 2 | 3 | I | (53) | 0 | 0 | N/C | (119) |
| | 0 | 3 | I | (114) | 1 | 1 | N/C | (17) |
| | 4 | 4 | N/C | (24) | 2 | 0 | D | (64) |
| | 2 | 3 | I | (168) | 2 | 1 | D | (134) |
| | 3 | 4 | I | (41) | 1 | 0 | D | (218) |
| | 0 | 1 | I | (31) | 0 | 0 | N/C | (63) |
| | 1 | 2 | I | (21) | 0 | 0 | N/C | (14) |
| | 1 | 4 | I | (49) | 1 | 1 | N/C | (18) |
| | 2 | 2 | N/C | (21) | 3 | 0 | D | (21) |
| | 1 | 2 | I | (35) | 4 | 1 | D | (14) |
| | 0 | 0 | N/C | (35) | 0 | 0 | N/C | (35) |
| | 0 | 2 | I | (62) | 2 | 3 | I | (35) |
| | 1 | 1 | N/C | (28) | | | | |
| Median | 1 | 3 | | (45) | 1 | 0 | | (37) |

Responders specimen 1 *vs.* 2, $P>0.05$, Non-responders specimen 1 *vs.* 2, $0.05>P>0.002$ (Wilcoxon paired analysis).

D, I and N/C = decrease, increase and no change respectively.

Values in parenthesis are the number of days between specimens 1 and 2.

Specimen 1 was obtained within 14 days of starting chemotherapy.

in the median grading of both groups, but after a period of therapy the F-AT levels were very different. In the non-responders, 75% of the women had significantly higher levels of F-AT in their second specimen than in the first specimen ($0.05>P>0.02$; paired Wilcoxon). In the responders, the F-AT level was either very low in both specimens or was lower in the second specimen; but this difference in expression was not statistically significant ($P>0.05$). F-AT was undetectable (grade = 0) in 8/14 of the responders when measured on a second occasion (see Table I).

Very low levels of F-AT were also detected in some sera from age-matched healthy individuals (Figure 4). In a group of 9 women (only 6 shown on the figure), individuals were graded as either 0 or 1. For the purposes of comparison, a serum extract from a patient with a recurrent ovarian cancer was also separated with the 'healthy' serum extracts.

Figure 5 shows the serum F-AT levels in 8 patients who were monitored at various times throughout their chemotherapy. All these patients responded to chemotherapy, had a period of clinical remission, followed by recurrence of tumour growth. To allow comparison between patients, F-AT expression is given as a grading. The patients are ordered according to the staging of their disease and the amount of tumour removed at laparotomy ('C' most of the tumour remained; 'B' >50% of the tumour removed). With patients (e–h) the presence of liver metastases were noted on the surgical report ('L'). The electrophoretic patterns for three of the patients (e, g and h) have already been shown in Figures 1 and 2. For all the patients investigated in this way, the pattern of change was similar to that previously described, i.e. low, falling or undetectable levels of F-AT in remission and higher or rising levels during recurrence. The pattern of F-AT expression was not correlated with the stage of the disease or the presence of residual tumour after laparotomy. Also, patients who had liver metastases did not have higher levels of F-AT when they had a recurrence of tumour growth. In five patients, F-AT fell to undetectable levels during remission, and in half the patients, increases in F-AT predated any clinical symptoms of tumour recurrence.

**Figure 4** The electrophoretic separations of 10 μl serum extract from six healthy women (lanes 1–6) and one patient with recurrent ovarian cancer (lane 7).

**Figure 5** An assessment of the expression of F-AT in serum extracts from 8 women who were undergoing treatment for ovarian cancer. The intensity of the F-AT is given as an arbitrary grade. The presence of clinically-detectable tumour and the time after start of chemotherapy are shown below the figure respectively. If F-AT was undetectable this is shown as a *. The Roman numerals indicate the stage of the disease at the start of therapy and the proceeding letter indicates the amount of tumour removed at laparotomy ('B' >50% removed; 'C' most of the tumour not removed). The superscript 'L' indicates the detection of liver metastases.

### Discussion

The abnormal forms of AT we isolated from the blood of ovarian cancer patients was a surprising finding because they appeared to be only present in high amounts when there was a recurrence of tumour growth. This suggested that they may be useful for predicting patient response to therapy. This was shown to be the case, but in order for them to have any real predictive value, it was necessary to measure F-AT in two blood specimens. One specimen to be collected at the start of therapy and the other specimen at a later date. Using this approach the majority of responding and non-responding patients could be discriminated. This discrimination would probably be made even more complete if F-AT was estimated in more than two specimens, and specimens could have been collected prior to the start of therapy.

The source of F-AT is unknown, the liver is the most likely candidate, as this organ is the major site for the synthesis of AT under normal circumstances (Laurell & Jeppsson, 1975). The presence of liver metastases could affect AT synthesis. Abnormally glycosylated AT has been shown to be synthesized by human hepatoma cells in culture (Carlson *et al.*, 1984). The situation is still unclear, because the presence of liver metastases did not seem to effect F-AT levels. Other sources of F-AT are possible, including the tumour itself, but we have so far failed to detect any AT in cultured ovarian cancer cells (unpublished observations).

Although the F-AT levels in the healthy sera used in this study were either very low or undetectable, we have also found in other studies, that younger women (median age = 37 y) have slightly higher levels (grades 1–2). As the majority of the women we used were post-menopausal, this suggests that F-AT could be affected by oestrogen levels. It is known that these substances can elevate total AT levels in the blood (Laurell & Rannevik, 1979). Further work is necessary to clarify this aspect.

Without further investigations it is difficult to understand the nature of the change that is occurring in AT in cancer. Lotus specificity is directed towards fucose that is linked in either a α-(1–2) position in sub-terminal galactose or in a α-(1–6) position to a N,N′diacetylchitobiose core (Petryniak & Goldstein, 1986). In early work, fucose was reported to be present in AT in very low amounts (Heimburger *et al.*, 1964; Crawford, 1973; Chan, Luby and Wu, 1973), but in more recent investigations, this finding has not been confirmed (Mega *et al.*, 1980; Hodges & Chan, 1982). The precise nature and number (3 or 4) of the carbohydrate sidechains on AT is also controversial (Mega *et al.*, 1980; Hodges & Chan, 1982; Bayard *et al.*, 1982). All studies find bi- and tri-antennary oligosaccharides and some have reported the absence of sialic acid on some units (Hodges & Chan, 1982; Bayard *et al.*, 1982; Hercz, 1984). The possibility that fucose could be added to existing sidechains of AT in cancer is not unfeasible.

The nature of F-AT varied from sera to sera, sometimes it was one diffuse band at 58 kD; whereas at other times there were two bands 56 and 58 kD. Non-extracted AT has reportedly a $M_r$ of between 50 and 55 kD (Laurell & Jeppsson, 1975), we have found an average $M_r$ of 55 kD for AT using our SDS/PAGE system (unpublished observations). The higher $M_r$ of F-AT is probably an overall reflection of changed AT glycosylation (e.g. increased glycans or increased branching), of which increased fucosylation is one part of this process. As well as increasing the absolute $M_r$, these changes could also decrease the amount of SDS bound, so reducing the electrophoretic mobility and increasing the apparent $M_r$. The abnormally-glycosylated AT found by Carlson *et al.* (1984) had a higher $M_r$ (56 kD).

It seems likely that the difference in F-AT between 'responders' and 'non-responders' reflects differences in tumour progression rather than differences in tumour growth, because both groups had similar amounts of tumour at the start of chemotherapy. In the 'non-responders', this change in tumour properties has already occurred before the start of treatment; whereas in the 'responders', it occurs when the tumour regrows. The ability to spread to the liver would seem to be the most likely property that the tumour could acquire to affect AT synthesis, but as already stated, there is no evidence to link F-AT levels and liver metastasis. Also, if the presence of tumour in the liver is affecting protein glycosylation, it is difficult to explain why the levels of fucosylated haptoglobin (F-Hp) are related to tumour burden (Thompson & Turner, 1987*b*) and not tumour progression. Possibly there are further changes in the properties of the tumour when it is well established in the liver, and this could explain the discrepancies between F-AT and F-Hp.

The predictive value of serum F-AT level could make it very useful in the routine biochemical screening of ovarian cancer. All current markers for this disease, including fucosylated haptoglobin, are only indicators of tumour burden, confirming an already established clinical diagnosis. Measurement of F-AT could greatly improve this situation and allow clinicians to make earlier changes in their therapeutic strategy, so improving patient survival and reducing the costs of patient care. Much further work is required to investigate the general applicability of this test to different treatment regimes, to the interference by non-cancerous conditions and to cancers arising in other sites. In addition, a method is being developed to measure F-AT using lectin bound to multi-well plates. This will considerably speed-up the assay and so make it more attractive for using in a routine hospital laboratory.

We gratefully acknowledge the hospital staff in the Newcastle upon Tyne area for assistance in obtaining the blood specimens and the North of England Cancer Research Campaign and the GO fund for financial support.

### References

BAYARD, B., KERCKAERT, J.-P., LAINE, A. & HAYEM, A. (1982). Uniformity of glycans within molecular variants of $\alpha_1$-protease inhibitor with distinct affinity for concanavalin A. *Eur. J. Biochem.*, **124**, 371.

BLAKE, M.S., JOHNSON, K.H., RUSSEL-JONES, G.J. & GOTSCHLICH, E.C. (1984). A rapid, sensitive method for detection of alkaline phosphatase-conjugated anti-antibody on Western blots. *Anal. Biochem.*, **136**, 175.

CARLSON, J., ERIKSSON, S., AIM, R. & KJELLSTROM, T. (1984). Biosynthesis of abnormally glycosylated $\alpha_1$-antitrypsin by a human hepatoma cell line. *Hepatology*, **4**, 235.

CHAN, S.K., LUBY, J. & WU, Y.C. (1973). Purification and chemical compositions of human $\alpha_1$-antitrypsin of the MM type. *FEBS Lett.*, **35**, 79.

CRAWFORD, I.P. (1973). Purification and properties of normal human $\alpha_1$ l-antitrypsin. *Arch. Biochem. Biophys.*, **156**, 215.

EVANS, A.S., DOLAN, M.F., SOBOCINSKI, P.Z. & QUINN, F.A. (1974). Utility of serum protein-bound neutral hexoses and L-fucose for estimation of malignant tumour extension and evaluation of efficacy of therapy. *Cancer Res.*, **34**, 538.

GUTHRIE, D. (1979). The treatment of advanced cystadeno-carcinoma of the ovary with gestronal and continuous oral cyclophosphamide. *Br. J. Obstet. Gynaecol.*, **86**, 497.

HEIMBURGER, N., HEIDE, K., HAUPT, H. & SCHULTZE, H.E. (1964). Study of the building stones of human serum proteins. *Clin. Chim. Acta*, **10**, 293.

HERCZ, A. (1984). The oligosaccharides of human $\alpha_1$-antitrypsin. *Can. J. Biochem. Cell Biol.*, **62**, 19.

HODGES, L.C. & CHAN, S.-K. (1982). Locations of oligosaccharide chains in human $\alpha_1$-protease inhibitor and oligosaccharide structures at each site. *Biochemistry*, **21**, 2805.

LAURELL, C.-B. & JEPPSSON, J.-O. (1975). Protease inhibitors in plasma. In *The Plasma Proteins*, Putnam, F.W. (ed) **1**, p. 229. Academic Press: New York.

LAURELL, C.-B. & RANNEVIK, K. (1979). A comparison of plasma protein changes induced by danazol, pregnancy and estrogens. *J. Clin. Endocrinol. Metab.*, **49**, 719.

MEGA, T., LUJAN, E. & YOSHIDA, A. (1980). Studies on the oligosaccharide chains of human $\alpha_1$-protease inhibitor. *J. Biol. Chem.*, **255**, 4057.

PETRYNIAK, J. & GOLDSTEIN, I.J. (1986). Immunochemical studies on the interaction between synthetic glycoconjugates and α-L-fucosyl binding lectins. *Biochemistry*, **27**, 2829.

THOMPSON, S. (1987). Simplified and reproducible silver staining of proteins in polyacrylamide gels. *Med. Sci. Res.*, **15**, 1253.

THOMPSON, S., GUTHRIE, D. & TURNER, G.A. (1987*a*). Two abnormally fucosylated proteins in cancer sera: Markers of tumour burden and of response to therapy. *Br. J. Cancer*, **56**, 189.

THOMPSON, S., LATHAM, J.A.E. & TURNER, G.A. (1987*b*). A simple, reproducible and cheap batch method for the analysis of serum glycoproteins using Sepharose-coupled lectins and silver staining. *Clin. Chim. Acta.*, **167**, 217.

THOMPSON, S. & TURNER, G.A. (1987*a*). Abnormally fucosylated haptoglobin in cancer sera. *Br. J. Cancer*, **55**, 348.

THOMPSON, S. & TURNER, G.A. (1987*b*). Elevated levels of abnormally-fucosylated haptoglobins in cancer sera. *Br. J. Cancer*, **56**, 605.

TRACY, R.P., CURRIE, R.M. & YOUNG, D.S. (1982). Two-dimensional gel electrophoresis of serum specimens from a normal population. *Clin. Chem.*, **28**, 890.

TURNER, G.A., ELLIS, R.D., GUTHRIE, D., LATNER, A.L., ROSS, W.M. & SKILLEN, A.W. (1982). Cyclic GMP in urine to monitor the response of ovarian cancer to therapy. *Br. J. Obstet. Gynaecol.*, **86**, 760.

TURNER, G.A., SKILLEN, A.W., BUAMAH, P. & 4 others (1985). Relationship between raised concentrations of fucose, sialic acid, and acute phase proteins in serum from patients with cancer: Choosing suitable serum glycoprotein markers. *J. Clin. Pathol.*, **38**, 588.

WAALKES, T.P., MROCHEK, J.E., DINSMORE, S.R. & TORMEY, D.C. (1978). Serum protein-bound carbohydrates for following the course of disease in patients with metastatic breast carcinoma. *J. Natl Cancer Inst.*, **61**, 703.